# 

**Published:** 1888-12-08

**Authors:** 


					TTy /), W ' i ? U
PERMANENTLY ENLARGED TO 32 PAGES.
PRICE ONE PENNY.
tto. 115, Vol. V-
-December 8th, 1888.
Per Annum, 6s. 6d., post free.
Monthly Parts. 6d.; post free, 7id.
Ditto per Ann. 7s. 6d.j post free.
IVegUtered at the General Post Oillce ML" <m JMC?    -
a? a Newspaper.3 , ^ r~^ Ditto per Ann. 7s. 6d., post free.
A Weekly Institutional Journal of
Science, /Iftebichtc, IRutsing, mxb flbijUautljrcips.

				

